# Notes

**Published:** 1884-07

**Authors:** 


AT a recent meeting of the Philadelphia County Medical Society, Dr. Carl Seiler called attention to the value of tincture of benzoin in the treatment of chapped hands and frosted feet. He has used it in a number of cases with much success. It is applied by simply painting it on the skin. The stocking may be prevented from sticking to the feet by rubbing some oil over the benzoin.—*polyclinic.*

DEATH FROM CHLORAL.—The *Medical Press* reports the death of Dr. John Middleton, at Stockton-on-Tees, England, from an overdose of chloral. The deceased, a retired army surgeon, had been the subject of fatty degeneration of the heart, kidneys, and liver (as proved by a post-mortem), and took the drug for the relief of wakefulness. He was immediately aware of his misadventure, and called his housemaid, to inform her of his danger. He soon became unconscious, and died before skillful aid could be had. A sad feature of the case is that the deceased had three motherless children, and that, though in precarious health, the one desire of his life was to live till his children were grown and settled in life. Is it not high time that doctors in ill health should fully understand the danger of taking this vile drug in indefinite doses, and refrain from its use except upon the advice of a medical attendant? Deaths from chloral are too common in these days.—*Louisville Medical News.*

---

SELLING HIS PRACTICE.—A physician sold his practice and agreed not to practice "in that city or its vicinity." He broke this agreement, and his successor brought suit for an injunction, which was granted to him generally. The case was carried to the Supreme Court of Michigan, where the decree was confirmed. The court defined the term "city or its vicinity" to include the territory surrounding the city for the distance of ten miles from its corporate limits. "The extent of territory, in the matter of doing business, included in the term 'vicinity of the city,' must necessarily depend in a great measure upon the size of the city, its location and particular surroundings."—*Lancet and Clinic.*

